# Supplementary material for: Proteomic profiling of the local and systemic immune response to pediatric respiratory viral infections
Source: mSystems. 2024 Nov 29;10(1):e01335-24. doi: 10.1128/msystems.01335-24 (PMC11748518; doi:10.1128/msystems.01335-24)
Supplement: Supplemental Material — Supplemental methods, table, and figures. [file msystems.01335-24-s0001.docx]

**SUPPLEMENTARY METHODS, TABLES, AND FIGURES**

**Title:** Proteomic profiling of the local and systemic immune response to pediatric respiratory viral infections

**SUPPLEMENTARY METHODS**

*Criteria for enrollment in cohort*

Inclusion:

- Aged 31 days to 18 years.
- Expectation of requiring mechanical ventilation (MV) via endotracheal tube for at least 72 hours.

Exclusion:

- Tracheal aspirate (TA) sample not obtained within 24 hours of intubation
- Tracheostomy in place, or plans to place one
- Conditions in which deep tracheal suctioning was contraindicated
- A previously episode of mechanical ventilation during the hospitalization
- Limitations of support
- Prior enrollment in the study

*Adjudication of LRTI status*

Viral LRTI Criteria:

- Admitting diagnosis of lower respiratory tract infection, AND
- Concordant retrospective clinical diagnosis made by study site clinicians, integrating clinical, laboratory, microbiology, and radiology data at the end of admission, AND
- Positive microbiologic test for a respiratory virus, taking place -72 hours to +48 hours of intubation, EITHER
  - Clinical microbiologic testing (respiratory pathogen or respiratory viral PCR panel) from nasal swab, tracheal aspirate, or bronchoalveolar lavage, OR
  - Tracheal aspirate metagenomic next generation sequencing
  - The following viruses were considered respiratory viruses: adenovirus, coronavirus, cytomegalovirus, enterovirus, human metapneumovirus, influenza A/B/C, parainfluenza 1/2/3/4, rhinovirus, respiratory syncytial virus (RSV).

Bacterial/Viral Coinfection Criteria

- All of the above, AND
- Detection of a bacteria capable of causing lower respiratory tract disease, taking place -72 hours to +48 hours of intubation, EITHER
  - Clinical microbiologic testing from sputum or bronchoalveolar lavage culture, OR
  - Clinical respiratory pathogen PCR panel from nasal swab, tracheal aspirate, or bronchoalveolar lavage, OR
  - Tracheal aspirate metagenomic next generation sequencing

No LRTI Criteria

- Non-infectious admitting diagnosis
- Concordant retrospective clinical adjudication made by study site clinicians, integrating clinical, laboratory, microbiology, and radiology data at the end of admission
- No pneumonia diagnosis during admission, including ventilator-associated pneumonia
- No positive clinical microbiologic test for a respiratory viral pathogen (not all patients in this group had comprehensive clinical microbiologic testing)

**SUPPLEMENTARY TABLES**

|  | Coefficients | | | | | |
| --- | --- | --- | --- | --- | --- | --- |
| Protein | Fold 1 | Fold 2 | Fold 3 | Fold 4 | Fold 5 | Complete model |
| (Intercept) | -2.167 | -12.469 | 0.894 | -13.998 | -0.526 | -1.793 |
| BAFF |  |  |  | 0.829 |  |  |
| BTC |  |  | -0.080 | -0.165 |  |  |
| CDK2/cyclin A | 0.029 |  | 0.083 |  | 0.037 | 0.016 |
| CLC1B | -0.117 |  | -0.134 |  |  |  |
| EphB6 |  | -0.028 |  |  |  |  |
| ERBB1 |  |  |  |  | -0.579 |  |
| FABP | -0.234 | -0.046 | -0.244 | -0.129 | -0.389 | -0.232 |
| Factor D |  | -0.071 |  |  |  |  |
| FBLN3 |  | -0.066 | -0.432 |  |  | -0.088 |
| FGF-12 |  | 0.062 |  |  |  |  |
| GPVI |  | -0.310 |  | -0.138 |  | -0.179 |
| Granulysin | 0.032 | 0.555 | 0.436 | 0.083 | 0.832 | 0.344 |
| Granzyme B | 0.106 |  | 0.012 | 0.235 | 0.052 | 0.077 |
| Hemopexin |  |  | -0.052 |  |  |  |
| Heparin cofactor II |  |  |  |  | -0.013 |  |
| IgD |  |  |  | -0.071 |  |  |
| I-TAC | 0.080 |  |  |  |  |  |
| IGFBP-7 | -0.104 |  |  |  |  |  |
| IL-1 sRII |  |  |  |  | 0.010 |  |
| IL-1b |  |  |  |  | -0.023 |  |
| ISG15 | 0.600 | 0.273 | 0.372 | 0.357 | 0.392 | 0.351 |
| KIF23 | 0.006 | 0.159 | 0.227 |  | 0.056 |  |
| Marapsin |  | 1.134 |  |  |  |  |
| MDC |  | -0.056 |  |  | -0.040 |  |
| MICA |  | -0.006 |  |  |  |  |
| MIP-5 | -0.144 |  |  |  |  |  |
| NAP-2 |  | -0.276 | -0.024 |  | -0.084 | -0.035 |
| Soggy-1 |  |  |  | 0.585 |  |  |
| VEGF-D |  | -0.061 |  | -0.144 |  |  |

**Table S1: Protein weights for the LASSO logistic regression model for distinguishing between vLRTI and No LRTI.** Weights for each individual fold in the five-fold cross validation are shown, as well as the complete model (nine proteins) incorporating all of the patients.

**SUPPLEMENTARY FIGURES**


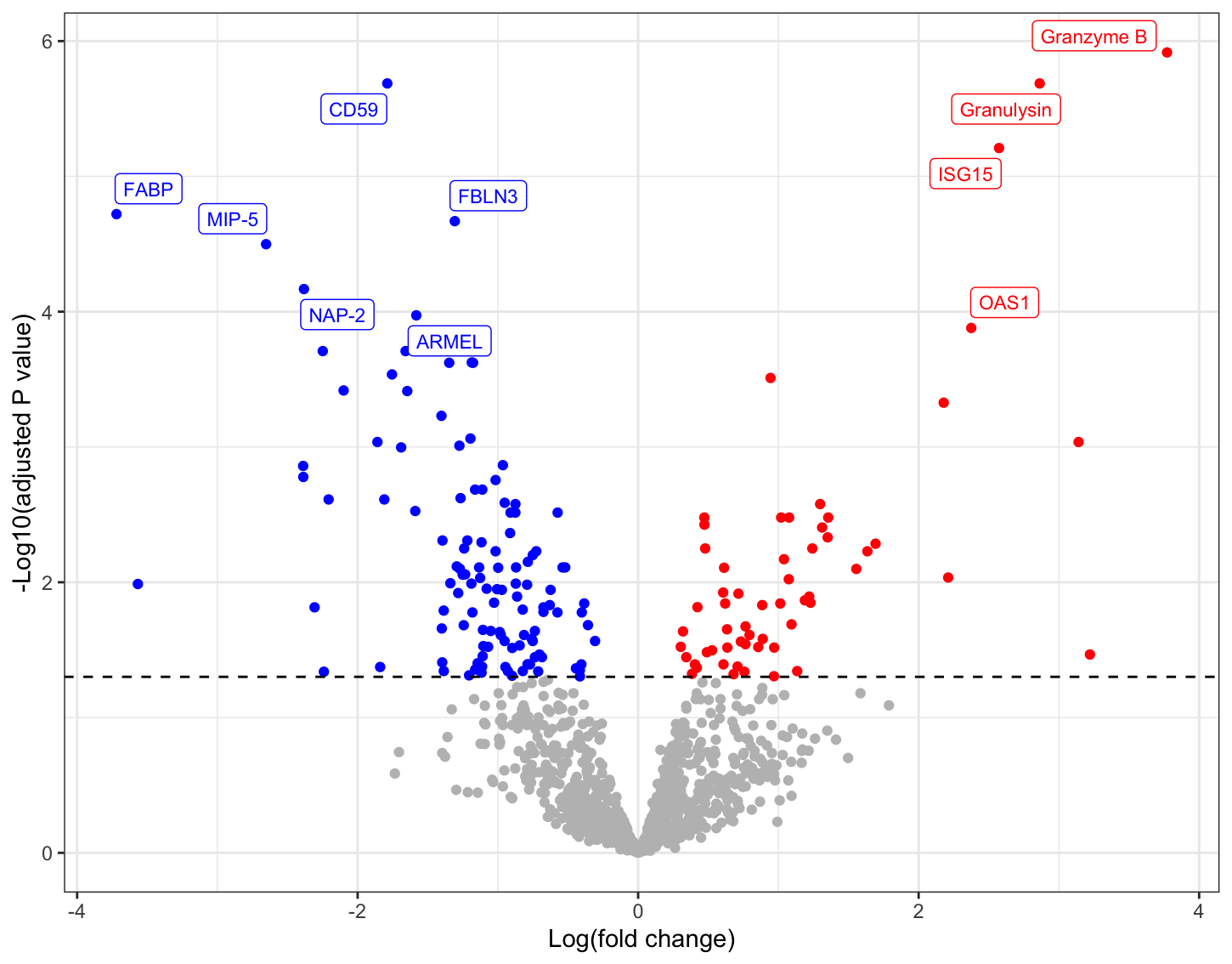


**Figure S1: Age-adjusted comparison of host protein expression between vLRTI and No LRTI cohorts in tracheal aspirate.** Proteins significantly upregulated in vLRTI are shown in red and proteins significantly downregulated in vLRTI are shown in blue. The top 10 proteins based on adjusted P-value are labeled.


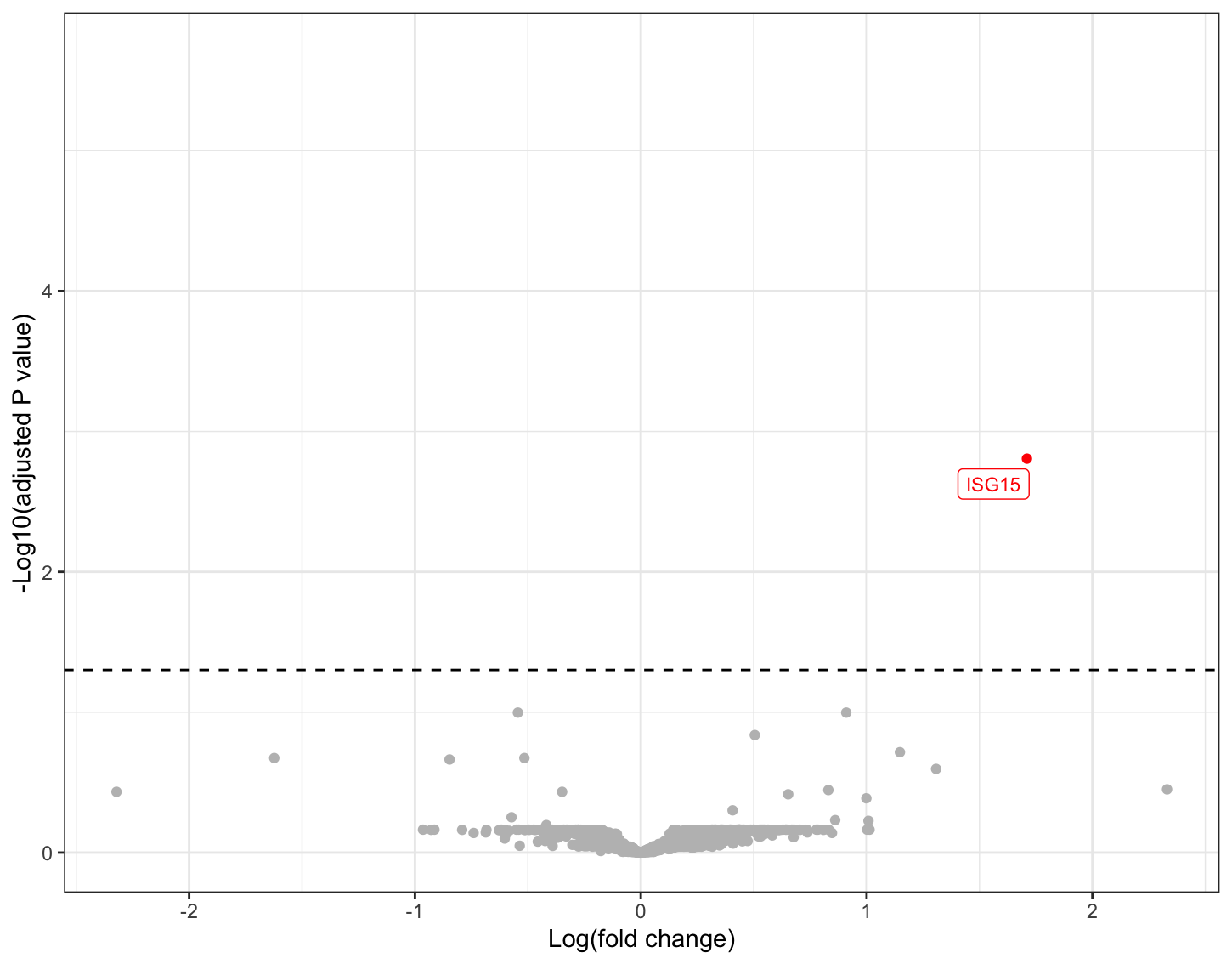


**Figure S2: Age-adjusted comparison of host protein expression between vLRTI and No LRTI cohorts in plasma.** After age adjustment, only one protein, ubiquitin-like ISG-15, remained significantly different between the groups.


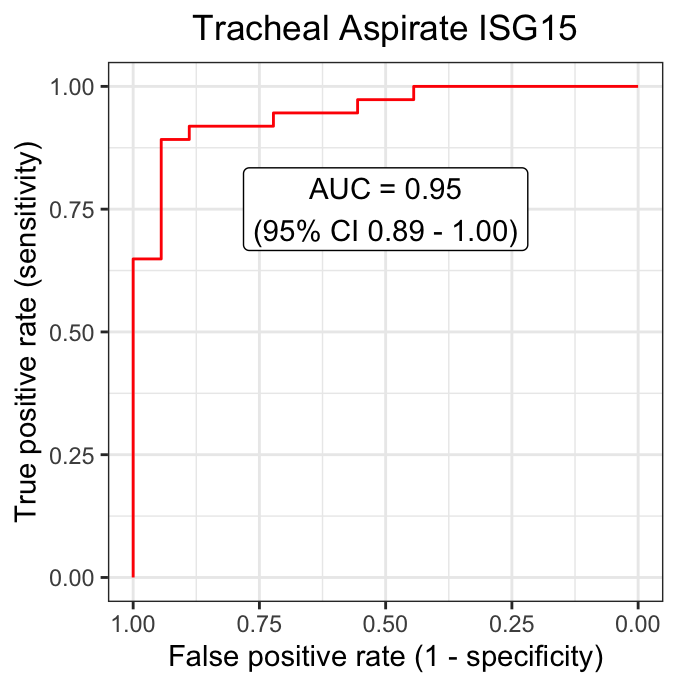

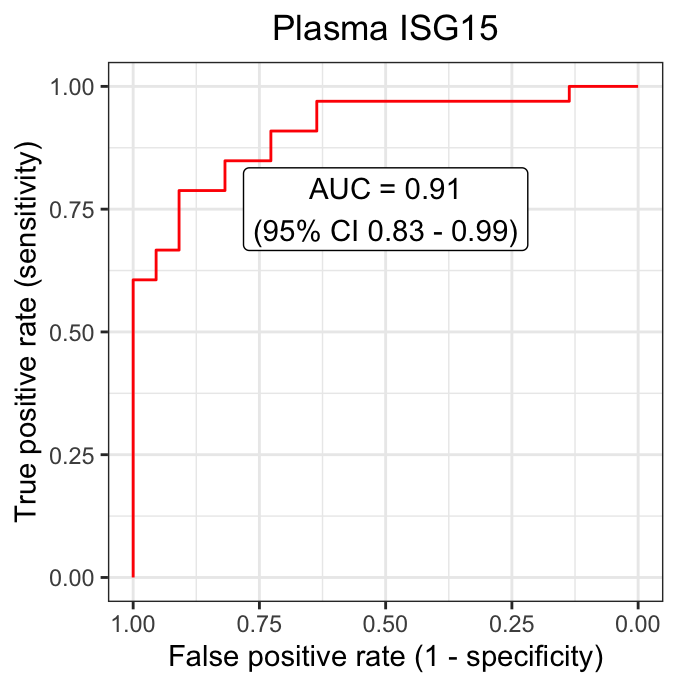


**Figure S3: Reciever operating characteristic curve for tracheal aspirate and plasma ISG-15.** Performance characteristics for the ISG-15 (log base 2 of relative fluorescence units) for distinguishing between vLRTI and No LRTI were calculated separately for TA and plasma samples.
